# Knowledge, and practice of cervical cancer prevention and associated factors among commercial sex workers in Shashemene Town, West Arsi, Oromia Region, Ethiopia

**DOI:** 10.1186/s12905-022-01819-6

**Published:** 2022-06-16

**Authors:** Muche Argaw, Aynamaw Embiale, Belay Amare

**Affiliations:** 1 Department of Midwifery, College of Medicine and Health Science, Wolkite University, Wolkite, Southern Ethiopia Ethiopia; 2 Department of Midwifery, College of Medicine and Health Science, Madda Walabu University, Bale Goba, Ethiopia; 3 Department of Midwifery, College of Medicine and Health Science, Hawassa University, Hawassa, Southern Ethiopia Ethiopia

**Keywords:** Cervical cancer, Knowledge, Prevention, Screening

## Abstract

**Background:**

The distribution of deaths and morbidities related to cervical cancer is disproportionally higher in low- and middle-income countries. In Ethiopia, there is a limited study on cervical cancer prevalence on Sex Workers, but a study conducted in Central America risk of developing HPV infection in sex workers is 2.5 times more than the general population. But a study conducted in the general population in Ethiopia reports that the incidence and mortality due to cervical cancer in Ethiopia is 26.4 and 18.4/100,000 respectively. However, there is limited data on knowledge and practice of cervical cancer prevention among sexual sex workers in Ethiopia.

**Methods:**

A health institution-based cross-sectional study design was used to investigate the knowledge and practice of cervical cancer prevention and its associated factors among 405 women of commercial sex workers with a systematic random sampling technique and with an interviewr administered. Multivariate logistic regression was used to identify associated factors of knowledge and practice towards cervical cancer prevention with a p value < 0.05.

**Result:**

In this study, three hundred eighty-five participants were included making a response rate of 95%. About half (50.1%) of respondents have knowledge regarding to cervical cancer, screening, and its prevention, and 20.3% of respondents were practiced cervical cancer screening. Having a history of use of combined oral contraceptives, AOR 2.190, (95% CI 1.374–3.492) and having a history of sexually transmitted infection, AOR 1.861, (95% CI 1.092–3.172). were significantly associated factors with knowledge of cervical cancer prevention. Regarding their uptake of cervical cancer screening, the level of knowledge was a significant factor, AOR 17.216 (95% CI 7.092–41.793).

**Conclusions:**

The study participants have an average knowledge of cervical cancer and its prevention and their practice was low as other women. Curtailing cervical cancer; through increasing their knowledge by integrating sexual and reproductive health services to cervical cancer screening clinics and equipping them with audiovisual materials that will increase their knowledge that end up with good uptake.

## Background

The main cause of cervical cancer is HPV infection which is sexually transmitted and The peak time of acquiring HPV infections for both female and male are shortly after becoming sexually active [1]. Cervical cancer is most common which an estimated 530,000 new cases every year and total coverage of 7.9% among all female cancers in the world. Almost 90% of the 270,000 deaths from cervical cancer in 2015 occurred in low and middle-income countries [2].

Regarding to the number of death in related to cervical cancer Africa is the largest number of death and Latin America, Caribbean, Asia were the next rank next to Africa. Cervical cancer in eastern, middle, and southern Africa, as well as Melanesia, is the second leading cause of cancer death in females [3]. WHO and UN reports show that Ethiopia is the 20^th^ next to japan with mortality of 14/10,000 due to cervical cancer in 2013 [4].

American obstetrics and gynecology recommended that the initiation of screening with pap smear at the age of 21 every three years interval until the age of 29 years followed by screening with pap smear and HPV testing every five years until the age of 65 [5]. In low and middle-income countries, the coverage of screening is very low, and alternative screening methods are required [6]. Ethiopia practiced cervical cancer screening between 30 and 49 years of age at least every three years based on WHO recommendation [7].

The family guidance association of Ethiopia is a non-governmental, non-profit organization with over 43 years of dedication in providing quality, broad-ranging reproductive health services in Ethiopia. In the country, FGAE is the association and network of 18 comprehensive reproductive health clinics and 28 multi-service youth reproductive facilities located across the country. It provides health promotive, and disease preventive services such as Antenatal, care, post-natal care, family planning, immunizations, and cervical cancer screening. For such an odd population it provides services with confidential clinics [8].

Limitation of specific studies on commercial sex workers women in Ethiopia but reports of cervical cancer screening from different lower and middle-income countries, such as Nepal, India, Uganda, Nigeria, and Ethiopia showed that the coverage of cervical cancer screening was less than 10% [9, 10]. Cervical cancer screening tests and HPV vaccination have been feasible alternative modalities for the prevention and control of cervical cancer. However, to maximize the effectiveness of this program in terms of reaching the more vulnerable population, commercial sex workers' knowledge and practice towards cervical cancer prevention and protocol service play a vital role in establishing reproductive health education programs. The aim of this study to assess knowledge and practice of cervical cancer among commercial sex workers women in West Arsi, Oromia region, Ethiopia, 2021.

## Methods and materials

### Study area and period

This study was conducted in Shashemene town in west Arsi Oromia regional state, South-East Ethiopia from June 01/2020 –January 01/2021. The town was located 273 km from Addis Ababa capital city of Ethiopia. Based on the 2007 census conducted by central statistical agency west Arsi zone had a total population of 1,964,038, of whom 973,743 are men and 990,295 women and about 272,084 were urban inhabitants. The data were collected from commercial sex workers attending family guidance associations of Ethiopia at Shashemene center in the above time period. The number of commercial sex workers visiting FGAE Shashemene center for 6 months (two-quarter reports) Shashemene town administration was 623.

#### Study design

An institutional-based cross-sectional study design was carried out among commercial sex workers attending FGAE Shashemene center, west Arsi zone, Oromia region, Ethiopia, 2021.

#### Source population

All commercial sex workers living in Shashemene Town, West Arsi zone, South-East Ethiopia.

#### Study population

All commercial sex workers visiting confidential clinics of the family guidance association of Ethiopian at Shashemene center during the data collection period.

#### Eligibility

All commercial sex workers women who visit at family guidance association of Ethiopia; Shashemene center during the study period were included whereas living less than six months of residence were excluded criteria.

#### Sample size determination

Sample size calculated by open epi-info version 3.5.1 based on a single proportion formula, with N = Z^2^Pq/d^2^ where: N denotes sample size, Z denotes standard normal deviate, a constant set at the 95% confidence interval, which is 1.96% and taken as 40% [11]. The final sample size including the non-response rate of 5% was 405.

### Sampling technique

Systematic random sampling method was used with every “K” = 1 interval estimated from the ratio of expected flow in the last six months 623 to the total sample size of study 405. The first two were selected by lottery method. The second was the first sample unit, and then the second sample was the fourth. In case if selected sample participants were five non-respondents, skipping to the next participants and continued with the fashion until the required sample size was gained.

### Data collection procedures and tools

A structured interviewer-administered questionnaire was used to collect data from study participants. The questionnaire was adapted from different related literature with modifications in line with the objectives of this particular study. Parts of questionnaires were four in type; including socio-demographic characteristics, reproductive and sexual characteristics, knowledge, and practices on cervical cancer prevention.

### Data quality management

The questionnaire was first prepared in English and translated to local language and back to English by the different translator to keep its consistency and reliability. Data quality was ensured by conducting a pretest on 5% [19] from the total sampling at Hawassa FGAE.

A day of training was being given to the data collectors and the supervisor on the data collection tool and sampling techniques. We held regular supervision during the data collection period. Validity and reliability test were also done in a prior full-scale survey. To assure validity the response was double-cheeked with each respondent and confirmatory factors were analysis was used to measure validity objectively. Scales instruments were subjected to Cronbach Alpha to check reliability, with Cronbach alpha was 0.78. To control the quality of data for analysis, cleaning data before entry, running frequency and percentages then, editing was done.

### Operational definitions

#### Knowledge

If the Participant responds less than 50% correctly from a total of 24 knowledge-related questions. The questions were categorized as having poor knowledge and greater than 50% having Good knowledge [12].

#### Cervical cancer screening uptake

The action of ever use of available cervical cancer screening service.

#### Cervical cancer screening

Steps taken to identify women with any form of cervical changes and those without any form of cervical changes using an available method of screening.

#### Cervical cancer screening practice

The action of ever use of available cervical cancer screening service.

#### Practice

In this study, practice refers to the way that the respondents used to prevent cervical cancer (screening).

#### Prevention

Actions directed to preventing cervical cancer and promoting health to reduce the need for secondary health care including early and regular diagnosis or screening for early treatment.

#### Statistical analysis

After individual data were checked the completeness, the questionnaire was coded, checked, cleared, and entered on Epi data 3.1 software and exported to SPSS 22 for further analysis. Multivariate binary logistic regressions were carried out to examine the independent variables associated with the outcome variable. The assumption of binary logistic regression, model fitness was checked using Hosmer and Lemeshow goodness of fit test statistics and it was fit variables with a p value < were considered statistically significant.

## Results

### Socio-demographic characteristics of participants

Three hundred eighty-five participants were included in the study made a 95% response rate. The mean age of participants was 29.3 ± 5.5 with minimum and maximum ages of 20 and 41 respectively. Almost 95% of participants have secondary education and above and their monthly income was less than 2999 Ethiopian Birr in one-third of the participants (Table 1).

**Table 1:** Distributions of commercial sex workers women by socio-demographic characteristics living in West Arsi, Oromia region, South-East Ethiopia, 2020

Variables N = 385	Frequency	Percent
*Age*		
Age 20–24	86	22.3
Age 25–29	137	35.6
Age > = 30	182	42.1
*Religion*		
Orthodox	183	47.3
Muslim	20	5.2
Protestant	169	43.9
Others*	13	3.4
*Martial status*		
Single	201	52.21
Divorced	133	34.55
Widowed	51	13.24
*Level of education*		
Primary (1–8)	18	4.7
Secondary (9–10)	167	43.4
Preparatory (11–12)	200	51.9
*Monthly income*		
≤ 2999 ETB	173	44.9
3000–5000 ETB	136	35.3
≥ 5001 ETB	76	19.7

^*^others; catholic, Adventist, ETB = Ethiopian birr

### Reproductive and lifestyle characteristics

The median age of participants in terms of their first menstrual period and first sexual intercourse were 14.0 ± 1.8 and 21.0 ± 3.4 respectively. Two-thirds of participants had regular menstrual flow and about 229 (59.5%) had a history of pregnancy. Almost 95% of participants were treated for STI in their lifetime (Table 2).

**Table 2:** Commercial sex workers women on reproductive and lifestyle characteristics living in West Arsi, Oromia region, South-East Ethiopia, 2020

Variables N = 385	Frequency	Percentage (%)
*Age for first sexual intercourse*		
Age ≤ 19	256	66.49
Age 20–24	129	33.51
*History of pregnancy*		
Ever had any pregnancy	229	59.5
Ever no pregnancy	156	40.5
*Age at first pregnancy(n* = *229)*		
Age ≤ 24	110	23
Age ≥ 25	118	36.5
*History of family planning(n* = *229)*		
Not used	181	47
Ever used	204	53
*Duration to used COC(n* = *204)*		
< 1 year	113	29.4
1–3 year	40	10.4
> 3 year	28	7.3
*History of STI (n* = *385)*		
No	22	5.7
Yes	363	94.3
*History of smoking(n* = *385)*		
Yes	346	89.9
No	39	10.1
*History alcohol use (n* = *385)*		
Yes	370	96.1
No	15	3.9

### Knowledge level of participants

Almost two-thirds (254) of the participants of the study had awareness of cervical cancer. Regarding their knowledge from 24 knowledge-related questions, their mean level of knowledge was 6.7 ± 4.8 with minimum and maximum values of 1 and 22 respectively. More than half of the study participants knew that HPV is the cause of cervical cancer and about sixty percent of participants knew cervical cancer is preventable (Table 3).

**Table 3:** Knowledge level of commercial sex workers women towards cervical cancer prevention living in, West Arsi, Oromia region, South-East Ethiopia, 2020

Variables	Frequency	Percentage (%)
*Hearing about cervical cancer*	254	66
Source of information (n = 254)		
Health professionals	95	24.7
From radio/TV	189	74.4
Others^(magazine)^	15	3.9
Knowledge about risk factors		
HPV infection	226	58.7
Family history	208	54
Smoking	197	51.2
Having early sex	112	29.1
Older age	102	26.5
Others*	84	21.82
Knowledge about symptoms and sign’s		
Foul vaginal discharge	111	28.8
Vaginal bleeding	109	28.3
Post-coital bleeding	78	20.26
Pelvic pain	36	9.35
Cervical ca is preventable?		
Yes	232	60.3
Appropriate age of screening		
As soon as sexually active	207	53.8
Age 30 and above	178	46.2
Frequency of screening		
Every 3–5 years	48	12.5
Others	337	87.5

Others* prolonged use of COC, STI, lack of hygiene, repeated abortion

### The practice of cervical cancer screening and reasons not to screen

In this study one-fifth (78), of the participants were screened. From this more than half of the participants were advised by health professionals followed by relatives/or friends 53.85% and 44.87% respectively. In the other case reasons for non-screened were reporting, I am not sick, I have issued more than this followed by I am afraid of positive results 37.46, 32.25, 28.34% respectively (Table 4).

**Table 4:** Commercial sex worker women responses to practice and related living in west Arsi, Oromia region, South-East Ethiopia, 2020

Variables	Frequency	Percentage
History of screening	78	20.3
Who/which advice from you for screened only? (N = 78) Multiple choice		
Health professionals	42	53.85
Relatives	35	44.87
Media	19	24.36
Reasons forever not screened? (N = 307) circle all applied		
I don’t have time	65	21.17
I am not sick	115	37.46
I have other more serious issues to worry about	99	32.25
I have not heard of it	69	22.48
I am afraid of positive results	87	28.34
Others*	43	14.01

Others*I am careless, fear of social stigma, I don’t consider it is important/lack of symptoms

### Factors affecting knowledge of cervical cancer prevention

Regarding determinants of knowledge of cervical cancer prevention; factors that have a p value of < 0.25 in the binary logistic regression were included in the final multivariate analysis. Besides this history of using combined oral contraceptives were 2.19 times more likely to have a good level of knowledge than non-users (95% CI 1.374–3.492), having history STI 1.861 times more likely to have a good level of knowledge compared with having no history of STI (95% CI 1.092–3.172) (Table 5).

**Table 5:** Bivariate and multivariate analysis of factors associated with knowledge of cervical cancer prevention among commercial sex workers living in West Arsi, Oromia region, South-East Ethiopia, 2020

Variables	Knowledge of cervical cancer prevention		OR (95% CI)	
	Yes	No	COR	AOR
*Age of participants*				
Age < 25 year	30	56	0.368 (0.214–0.6347)	0.742 (0.371–1.483)
Age 25–29	67	70	0.568 (0.416–1.041)	0.785 (0.754–1.297)
Age above 29	96	66	1	1
*Hx of marriage*				
No	39	79	1	1
Yes	113	154	2.761 (1.761–4.347)	0.836 (0.386–1.809)
*Age at first sex*				
Age < 21	100	123	1	1
No > = 22	93	69	1.658 (1.102–2.494)	1.930 (0.754–1.887)
*Hx of pregnancy*				
No	139	102	1	1
Yes	54	90	2.917 (1.911–4.454)	1.674 (0.872–3.172)
*Hx of COC*				
Yes	117	64	3.079 (2.030–4.669)	2.190 (1.374–3.492)**
No	76	128	1	1
*History of STI*				
Yes	157	119	2.675 (1.681–4.258)	1.861 (1.092–3.172)*
No	36	73	1	1

Hx., History; 1 = reference category; **p value 0.001; *p value 0.022; COR, crude odd ratio; AOR, Adjusted odd ratio; COC, combined oral contraceptives; OR, odd ratio; CI, confidence interval

### Factors affecting the practice of screening

In the binary logistic regressions factors affecting uptake of cervical cancer screening was age of participants, having a history of marriage, a number of sexual partners, and history of STI, history of smoking, and general knowledge with a p value of less than 0.25, whereas, in the multivariate analysis having a good level of knowledge were seventeen times more likely to practice cervical cancer screening than those having poor knowledge AOR = 17.216 (95% CI 7.092–41.793) (Table 6).

**Table 6:** Bivariate and multivariate analysis of factors associated with practices of cervical cancer screening among commercial sex workers living in, West Arsi, Oromia region, South-East Ethiopia, 2020

Variables	Practices on cervical cancer screening		OR (95% CI)	
	Yes	No	COR (95%CI)	AOR (95%CI)
*Age of participants*				
Age < 25	7	79	3.030 (1.263–7.269)	0.469 (0.170–1.293)
Age 25–29	29	108	3.950 (1.690–9.233)	0.978 (0.529–1.805)
Age > 29	42	120	1	1
*Hx of marriage*				
Yes	11	136	1	1
No	67	171	3.259 (1.652–6.429)	2.149 (0.719–6.422)
*Number of sexual partners*				
< 4	13	96	2.275 (1.197–4.325)	1.115 (0.521–2.388)
> = 4	65	211	1	1
*Hx of STI*				
Yes	31	173	1	1
No	47	134	1.957 (1.180–3.248)	0.985 (0.479–1.672)
*Hx smoking*				
Yes	20	136	1	1
No	58	171	2.306 (1.323–4.021)	0.705 (0.285–1.743)
*Level of knowledge*				
Poor knowledge	6	186	1	1
Good knowledge	72	121	18.446 (7.777–43.254)	17.216 (7.092–41.793)*

^*^p value < 0.001, 1 = Reference category, OR, odd ratio; COR, crude odd ratio; AOR, adjusted odd ratio

## Discussion

This study addressed baseline information on knowledge and practice of cervical cancer prevention and associated factors among women commercial sex workers in the Oromia region, South-East Ethiopia.

In this study, the mean (± standard deviations) knowledge was 6.7065 ± 4.822 with minimum and maximum of one and twenty-two respectively from knowledge questions. Half of the study participants (50.1%) scored at least the mean value (95% CI 44.7–55.6). this is in line with a study done in Hosanna town southern Ethiopia 53.7% [13]. However, it is higher than a study done in northern Ethiopia Gonder 31% [14], north-west Ethiopia Finote Selam 23.1% [15], in Gabonese (27%) [16]. African American women (27%) [17], and Addis Ababa 43.8% [14]. This might be due to the nature of the study population, sample size included time of the study, the way knowledge participants operationalized, and the study area. Furthermore, the population has great opportunities for gaining knowledge from volunteer counselors, HIV-positive communities, and those working on HIV testing counseling.

On the other hand, factors significantly associated with level of knowledge among women of commercial sex workers were, have history utilization of combined oral contraceptives was two times more likely knowledgeable than non-users.

This was consistent with a study conducted in Ethiopia, Bahar- Dar city [18], sexual worker women who had a frequency of health facility visits were 4.8 times more knowledgeable than their counterparts. This implies that sex workers women who had the chance to visit health facilities and counseling frequently would have a higher chance of getting information about cervical cancer prevention.

Furthermore, commercial sex workers who have a history of sexually transmitted infections were1.86 times more likely knowledgeable than those with no history of STI, which incorporates the finding of the other studies in Ethiopia, Mekele city [19] has a history of infection were, 1.64 times more knowledgeable than the counterpart. other studies in Ethiopia Bahar-Dar City [18] also support the study that having a history of STI were 6.9 times more knowledgeable than no history of STI.

This might be due to women who diagnose and treated of STI were the chance to get information and counseling about cervical cancer prevention and the mechanism of protecting themselves.

This study also revealed that the Practice of cervical cancer screening among women of commercial sex workers was 20.3% (95% CI 16.1–24.2) from 385 participants. This was higher than studies done in different lower and middle-income countries, such as Nepal, India, Uganda, Nigeria, Ethiopia showed that the coverage of cervical cancer screening was less than 10% [20]. The difference might be due to different sources of population, levels of knowledge, and sample size.

In the Ethiopian context, the report of a world health survey indicated that the national coverage of cervical cancer screening was 0.6% in Ethiopia [21]. In both facility and community-based Cross-sectional studies in a different region of Ethiopia, cervical cancer screening was generally low in Hawassa 11.4% [22], Mekele 10.7% [23]. Gurage zone, Butajira town 15.1% [24], Mizan-Tepi 14.83% [24], Arba Minch 9.6% [25], west gojam zone Finote Selam 7.3% [15], Hosanna Town 9.9% [13], and Addis Ababa 3.5% [14]. The difference might be different area, study population, and exposure level total sources of population.

In this study the only predictor of cervical cancer uptake was level of knowledge; having a good level of knowledge was 17.216 times more likely to practice cervical cancer screening. This is finding also supported by 5.9 times in Tanzania Dare-Selam [26] and seven times in Malawi. This possible explanation might be due to knowledge is the basic required performing any recommended practices to prevent cervical cancer and had the knowledge for a certain problem could be also removed misconceptions, and also have a better understanding to undertaking actions.

### Limitation

Cross-sectional study design which is causal- effect dilemma and institution-based not include out of the institution. Poor generalizations to the whole commercial sex workers in West Arsi zone, Oromia region, West–East Etiopia. The result was incomparable since previous studies were not on commercial sex workers.

## Conclusion

The study participants have an average knowledge of cervical cancer, its prevention, and their practice was low as other women. Curtailing cervical cancer through; increasing their knowledge by integrating sexual and reproductive health services to cervical cancer screening clinics and equipping them with audiovisual materials that will increase their knowledge. Better to integrated family planning and sexually transmitted infection treatment centers increase their knowledge of screening that end up with good uptake.

## Data Availability

The data sets used during the current study are available from the corresponding author on reasonable request.

## Abbreviations

AOR: Adjusted odds ratio
CI: Confidence interval
COC: Combined oral contraceptive
COR: Crud odds ratio
UN: United Nation
HPV: Human papillomavirus
WHO: World Health Organization
SPSS: Statistical Package for Social Science
STI: Sexually transmitted infection
FGAE: Family Guidance Associations of Ethiopia
